# Purification of aquaculture effluent using *Picralima nitida* seeds

**DOI:** 10.1038/s41598-022-26044-x

**Published:** 2022-12-14

**Authors:** Chinenye Adaobi Igwegbe, Prosper Eguono Ovuoraye, Andrzej Białowiec, Charles Odilichukwu R. Okpala, Okechukwu Dominic Onukwuli, Mohammad Hadi Dehghani

**Affiliations:** 1 Department of Chemical Engineering, Nnamdi Azikiwe University, P.M.B. 5025, Awka, 420218 Nigeria; 2 Department of Chemical Engineering, Federal University of Petroleum Resources, P.M.B. 1221, Effurun, Nigeria; 3 Department of Applied Bioeconomy, Wroclaw University of Environmental and Life Sciences, Wrocław, Poland; 4 Faculty of Biotechnology and Food Science, Wrocław University of Environmental and Life Sciences, 51-630 Wrocław, Poland; 5 UGA Cooperative Extension, College of Agricultural and Environmental Sciences, University of Georgia Athens, Athens, GA 30602 USA; 6 Department of Environmental Health Engineering, School of Public Health, Tehran University of Medical Sciences, Tehran, Iran; 7 Center for Water Quality Research, Institute for Environmental Research, Tehran University of Medical Sciences, Tehran, Iran; 8 Center for Solid Waste Research, Institute for Environmental Research, Tehran University of Medical Sciences, Tehran, Iran

**Keywords:** Environmental sciences, Chemistry

## Abstract

Aquaculture effluent treatment is essential to eliminate the undesirable characteristics of water to ensure cleaner production and environmental sustainability. In an effort to develop green coagulant without compromising cost, this research investigated the feasibility of aquaculture effluent (AQEF) pollutant removal using *Picralima nitida* seeds extract (PNSC) and its bio-coagulation/adsorption kinetic characteristics with the substrate in water. The coagulative decrease was observed in terms of TD (turbidity), TSS (total suspended solids), COD (chemical oxygen demand), BOD (biochemical oxygen demand), and COLR (color) from AQEF. The active coagulant was extracted from the seeds and analyzed for its spectral and morphological characteristics through FTIR and SEM. The influence of PNSC dosage (0.10–0.50 g L^−1^), pH (2–10), settling time (0–60 min), and temperature (303–323 K) on the removal of contaminants were surveyed. The process kinetics of coagulation–flocculation were also explored. Maximal TD reduction of 90.35%, COD (82.11%), BOD (82.38%); TSS (88.84%), and COLR (65.77%) at 0.2 g PNSC L^−1^, pH 4, and 303 K was achieved. Analysis of variance (ANOVA) tests proved that pH, temperature, and settling time had a significant effect on pollutant removal. Results fitted Von Smoluchowski’s perikinetics theory at the optimum conditions, which gave R^2^ > 0.900. At perikinetics circumstances, the *K*_*b*_ (reaction rate) and ${t}_{f\frac{1}{2}}$ (half-life) correspond to 0.0635 Lg^−1^ min^−1^ and 1.9 min. More so, sorption results fitted the Lagergren over the Ho model. Additionally, the net cost of using PNSC to handle 1 L of AQEF (including electricity, material, and labor costs) was evaluated to be €4.81. Overall, the PNSC appears reliable and useful in pretreating AQEF for improved biodegradability and superior effluent quality.

## Introduction

Fisheries and their resources constitute a wide source of food and feed a large part of the world’s population besides being a huge employment sector. Many aquaculture facilities produce large effluent volumes containing components such as suspended particles, nitrates, nitrites, nitrogen, ammonia, and total phosphorus^1–4^. These components are considered fish aquaculture pollutants, resulting in major environmental issues^1^ such as eutrophication and oxygen depletion^2^. The production of 1 tonne of channel catfish releases an average of 9.2 kg of nitrogen, 0.57 kg of phosphorus, 22.5 kg of BOD, and 530 kg of settleable solids into the environment^5,6^.

Aquaculture effluents have been managed using different treatment methods such as biodegradation^7–9^, coagulation^10–12^, oxidation^13,14^, filtration^15–17^, adsorption^18–20^, electrocoagulation^21–23^, or in constructed wetlands^24,25^ or even a combination of these methods^26^ prior either reuse or disposal. However, the majority of the above-mentioned treatment methods do produce sludge, require much higher energy, and depend on frequent maintenance^1^, which makes the development of effective and low-cost treatment imperative. Moreover, the fraction of settleable particles contains the bulk of the phosphorus emitted from intensive fish production 50–85%^27–29^. As a result, any technique that might improve the removal of such solids would equally contribute to reducing the total phosphorus discharge^30^. Given the dilute nature of most aquaculture wastes, however, coagulation–flocculation (CF) has not been widely used in the aquaculture business^12,31^. The increased use of recirculating systems makes this option more attractive^30^.

Coagulation is often accomplished by the addition of ions possessing an opposite charge to that of the colloids^32^. Cationic coagulants would supply electric charges and diminish the negative charge (zeta potential) of the colloidal particles, which will result in the creation of big particles (termed flocs). Several operating factors, which include pH, coagulant type and dose, turbidity, temperature, and mixing speed, can impact the effectiveness and efficiency of the coagulation process^32,33^. The pH at which coagulation occurs is the most crucial parameter for optimum coagulation and flocculation performance. pH influences colloidal surface charge, functional groups, natural organic matter (NOM) charge, dissolved coagulant species charge, and coagulant solubility^34^. The coagulant dose is determined by the treatment water chemistry, namely the pH, alkalinity, ionic strength, hardness, and temperature^35^. The chemistry of wastewater has a considerable impact on the polymer performance as well as choosing the kind of polymer for usage as a coagulant and flocculation aid; this usually necessitates testing with the intended waste stream, and the final selection of the best polymer is made^34^. Each coagulant has an ideal dosage that results in the highest turbidity reduction, which varies based on the starting turbidity of the water^36^.

Chemical coagulants, such as aluminum sulfate [Al_2_(SO4)_3_·18H_2_O], have been widely employed for water treatment, despite challenges like pH adjustment, a large quantity of sludge resulting in a high disposal cost, ineffectiveness in water level, and a high price charge^37,38^. Green coagulants have acquired reasonable acceptance because they avoid the formation of hazardous sludge; also, green coagulants are cost-effective, highly degradable, environmentally friendly, and have a low potential to create water with high pH following treatment^39^. They can be utilized for the treatment of drinkable water since they are non-toxic^40^. Natural polyelectrolytes include plant and animal-based coagulants. They are water-soluble high-molecular-weight polymers with groups that can be converted into polymer molecules of very charged ions; in other words, polymers with ionizable sites^41^. They are polymeric organic compounds composed of long polymer chains that enmesh in water^42^. Depending on the functional groups (–OH, –COOH, and –NH) present, cationic polyelectrolytes can act as coagulants, neutralizing opposing charges and allowing particles to settle quickly. The rate of agglomeration is comparable to the impact of aluminum sulfate in an aqueous media^11,43^.

Plant coagulants also have antimicrobial properties and thereby reduce the content of microorganisms capable of causing disease, which has led to the increased development of natural plant coagulants for water purification^41^. Of increasing interest is the *Picralima nitida*, cultivated largely in the native tropical aspects of Africa, and locally known as the akuamma plant in Nigeria. The dried seeds have therapeutic properties. Traditional African oral medicine uses these seeds. *Picralima nitida* extracts possess antibacterial, antipyretic, and anti-parasitic properties. The plant is useful in the treatment of a variety of diseases^44,45^. The use of *Picralima nitida* extracts in wastewater treatment is scarce. More so, there is limited published research on the use of *Picralima nitida* seeds either as a coagulant or adsorbent^46^. It’s imperative, therefore to assess the efficacy of a new bio-coagulant in wastewater treatment, which employs locally accessible materials to actualize a feasible and cost-effective approach.

With the extent of the authors’ extensive search on coagulation studies, no *Picralima nitida* extract use has been documented for use in the coagulation of aquaculture effluent. This is a significant unique aspect of the present examination. The current research work specifically investigated the efficacy of *Picralima nitida* seed extract as an active coagulant/flocculant in the treatment of aquaculture effluent. The bio-coagulant structure was observed using FTIR (Fourier transform infrared) and SEM (Scanning electron microscopy) spectroscopy for its morphological and spectral features. The percentage reductions of pollutants such as TD, TSS, COD, BOD, and color (COLR) from aquaculture effluent (AQEF) were tested utilizing a green coagulant, *Picralima nitida* seeds coagulant (PNSC). The PNSC and AQEF physicochemical properties were also reported. The coagulation–flocculation kinetics and particle temporal evolution were also investigated in the study. The kinetics of sorption was also investigated. Furthermore, the cost–benefit analysis of employing PNSC to treat 1 L of AQEF was performed to supplement this quest for cleaner aquaculture production and environmental sustainability.

## Materials and methods

### Schematic overview of the experimental program

**Figure 1:**
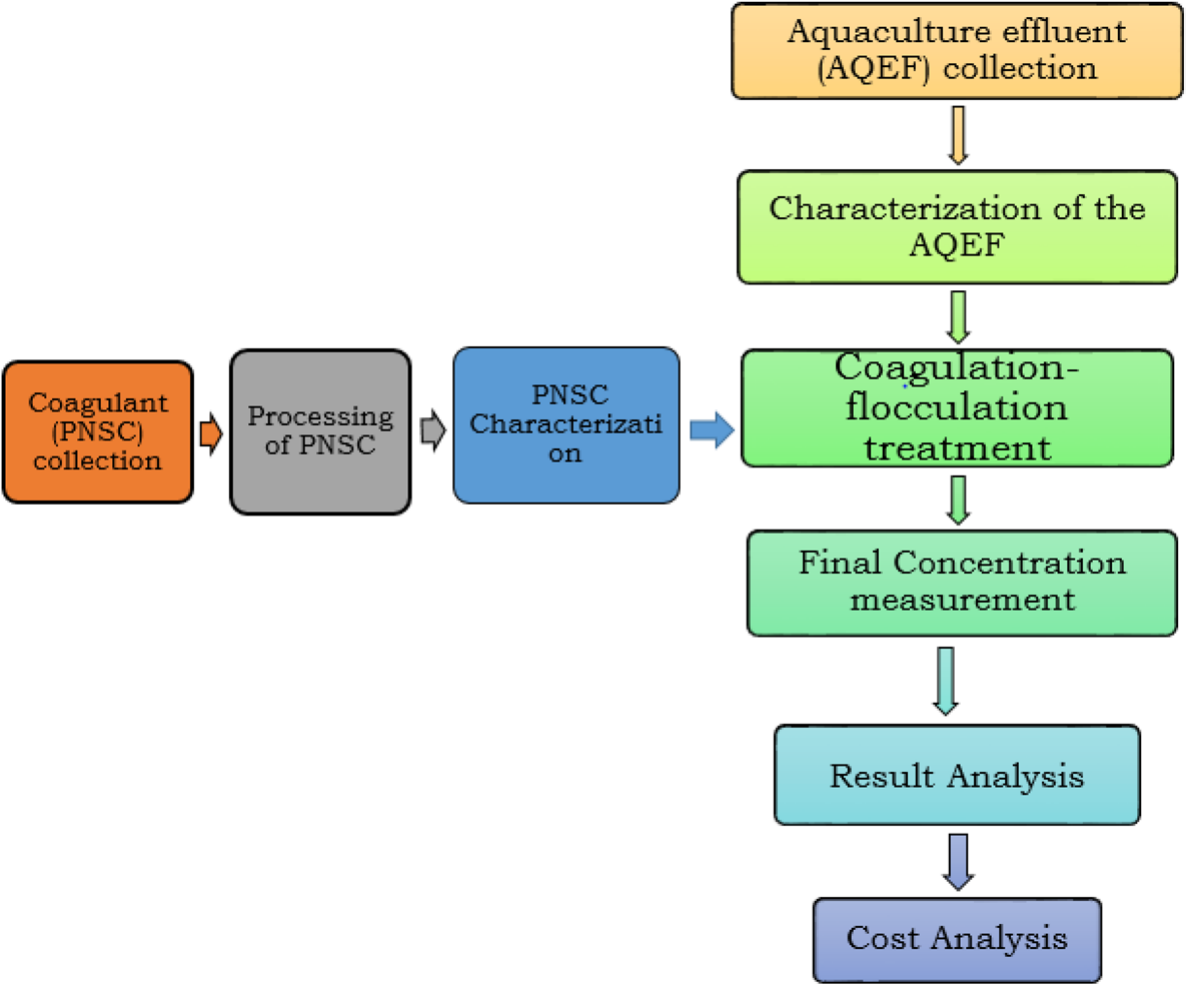
The schematic flow of the current research, from identification/collection of aquaculture effluent and *Picralima nitida* seeds, Jar test experiment, and final concentration measurement, before the result and cost analyses.

The schematic flow of the current research, from identification of aquaculture effluent (AQEF) characteristics, and *P. nitida* seeds, jars test experiment, and final concentration measurements, the result and cost analyses are shown in Fig. 1. For emphasis, this current work was performed to create a better understanding of the clarification efficacy of *Picralima nitida* seeds coagulant (PNSC) in managing aquaculture effluent, and to understand how the treatment system works. The authors engaged in both bio-coagulation and adsorption kinetics characterization. The chemicals used were of an analytical grade standard. All the conducted analytical measurements were performed independently, in adherence to relevant guidelines set out by the Department of Chemical Engineering, Nnamdi Azikiwe University, Awka, Anambra State, Nigeria.

### Source Identification/collection of the aquaculture effluent (AQEF) samples

**Figure 2:**
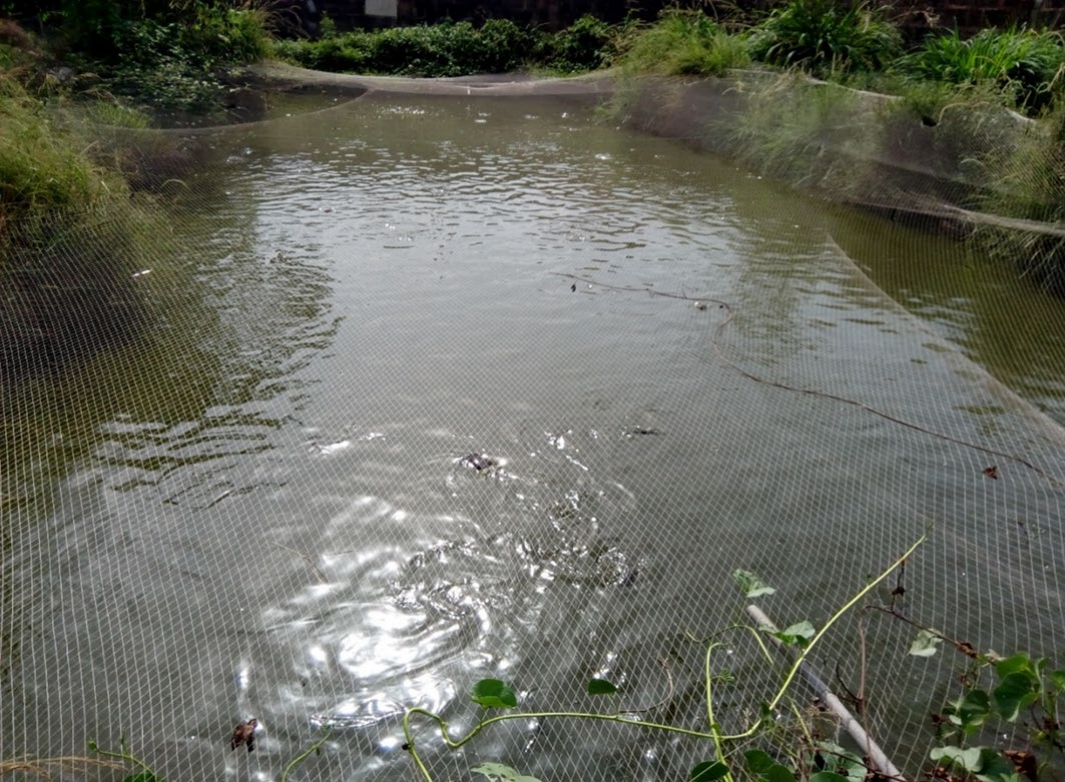
The collection point for AQEF (Latitude: 6^o^14′32.13″ N, Longitude: 7^o^06′16.44″ E). [This Figure was taken by "Chinenye Adaobi Igwegbe" during the research at point of wastewater collection].

The aquaculture effluent was collected from the water outlet of a local aquaculture facility (pond) located in Agu-Awka, Nigeria (Latitude: 6^o^14′32.13″ N, Longitude: 7^o^06′16.44″ E) (Fig. 2) using a gravity pump. The aquaculture source consisted majorly of a pond system (Over 10,000 capacity) based on catfish farming, typically grown for domestic consumption and commercial purposes. Catfish farming produces tons of wastewater required for treatment. The aquaculture wastewater and effluents usually contain high concentrations of organics, dissolved, and suspended solids considered point source pollutants. The physiochemical characteristic properties of the aquaculture effluent are shown in Table 1.

**Table 1:** The characteristic components of AQEF.

Parameter	Method	Value
Absorbance at 275 nm	–	0.842
Ammoniacal nitrogen	APHA 4110B^48^	0.829 mg L^−1^
Appearance	–	Green
BOD_5_	APHA 5210-B^48^	317 mg L^−1^
Biodegradability index (BI)	BOD_5_/COD	0.42
Calcium	APHA 3112B^49^	5.40 mg L^−1^
COD	APHA 5220-D^50^	758 mg L^−1^
Chloride	APHA 2510^50^	2.44 mg L^−1^
Conductivity	APHA, 2510B^50^	1963 µS cm^−1^
Iron	APHA 3112B^49^	0.425 mg L^−1^
pH	APHA 4500-H^48^	7.9
Potassium	APHA 3112B^49^	5.45 mg L^−1^
Temperature	APHA 2550 A^48^	303.2 K
TDS	APHA 2540 C^50^	650 mg L^−1^
Total phosphorus	APHA 4500-P C^48^	0.91 mg L^−1^
Total solids	APHA 2540 B^50^	695 mg L^−1^
Total suspended solids (TSS)	APHA 2540 D^50^	45 mg L^−1^
Turbidity (TD)	EPA Method 180.1^51^	404 NTU

The AQEF was preserved in jars at 277 K before treatments to avoid the defect of its characteristic components (Table 1) as determined by previously reported techniques^22,47^. All the conducted analytical measurements to manage the aquaculture effluent and to engage both bio-coagulation–flocculation performance of PNSC and kinetics characterization were in adherence to relevant guidelines set out by the Department of Chemical Engineering, Nnamdi Azikiwe University, Awka, Anambra State, Nigeria.

### Preparation and characterization of the PNSC

The usage of plant extracts instead of the entire plant is advantageous because it tends to prevent the growth of pathogenic organics and other additional pollutants^36^. The active coagulant was isolated from the coagulant precursor, Akuamma (*Picralima nitida*) seeds, to maximize the efficiency of contaminants removal. The Akuamma (*Picralima nitida*) seeds consisted of 16.0% ash, 28.4% protein, 0.17 g mL^−1^ bulk density, 7.40% fat, 10.5% fiber, 31.1% carbohydrate, and 46.6% moisture,were obtained from Ihembosi environs, Anambra state, Nigeria. This study complies with Nnamdi Azikiwe University’s institutional guidelines. The appropriate permission for the collection of plant specimens for experimentation was approved. The active coagulant was prepared according to the method described by Igwegbe et al.^46^. 30 g of the seeds was added to 250 mL of n-hexane at 70 °C for 6 h to extract the oil using an extractor. Prepared 250 ml salt solution of 4.0 g MgCl_2_, 25 g NaCl, 0.75 g KCL, and 1.0 g CaCL_2_ in 1000 mL of distilled water were mixed and shaken with 10 g of the *Picralima nitida* seeds at 323 K for 60 min. The filtrate of the mixture was hardened at 27 ± 2 °C.

The functional groups contained in the PNSC were identified to determine which chemical groups were present and participated in the coagulation experiment. PNSC (10 g) were freeze-dried for 24 h at 105 °C in a York Scientific Industries, India Lyophilizer, before their spectrum characteristics were investigated. The FTIR analysis was performed by combining the PNSC in a 1:100 ratio with dry finely powdered potassium bromide and collecting spectra from 4000 to 400 cm^−1^ at room temperature. The PNSC spectra were acquired using a Fourier transform infrared transmission system (Buck M520 Infrared spectrophotometer). SEM was performed via a Carl Zeis Analytical SEM Series. MA 10.EVO-10-09-49 to observe the surface morphology of the PNSC. The image was recorded for magnifications of 1000 × and 2000 × at a working distance of 15 mm and accelerating voltage of 15 kV using full BSD (backscattered electron dictator).

### Coagulation–flocculation test procedure

The standard jar test procedure conducted for the coagulation–flocculation treatment of AQEF was carried out using a flocculator (model ZSI-2120). The pH was measured using a pH meter (Hanna pH meter). To ensure a uniform concentration of the effluent medium, AQEF was agitated before collecting the samples for the experimentations. The effects of PNSC dose (0.10–0.50 g L^−1^), pH (2 to 10), settling time (0–60 min) and temperature (303–323 K) on TSS, COD, TDS, COLR, and BOD changes were investigated. The TD, TSS, COD, and BOD were tested using EPA Method 180.1^51^, APHA 2540 D^50^, APHA 5220-D^50^, and APHA 5210-B^48^, respectively. The COLR was tested by measuring the absorbance at 275 nm, which is the maximum wavelength obtained for AQEF^46^.

In the course of the jar test experimentation, a 500 mL measuring cylinder was used to measure the AQEF sample, which was then poured into various 1000 mL beakers. The pH was attuned using 1 M HCL or NaOH solutions. A 0.10 g L^−1^ dose of PNSC was added to each beaker, and the mixture was then agitated for 5 min at a stirring speed of 120 rpm, supported by 20 min of shaking at a reduced speed of 30 rpm at a temperature of 30 °C. The stirring was stopped to allow for studying the floc formed at various settling times (3–30) minutes. A syringe was used to extract 20 mL of the samples at 0.02 m depth from each of the 1000 mL beakers and tested for the coagulation efficiencies (%CGE) in terms of TD, TSS, BOD, COD, and COLR. The same procedure was repeated using other dosages of PNSC. In each case, the corresponding efficiencies (*%CGE*) were also evaluated following Eq. (1).

1 $$\% CGE = \left( {1 - \frac{{CGE_{f} }}{{CGE_{i} }}} \right) \times 100$$

${CGE}_{i}$ and ${CGE}_{f}$ are the initial and final concentrations of TSS, TDS, BOD, COD, and COLR respectively.

A one-way analysis of variance (ANOVA) test was performed to understand the significance and the effect of the changes in process parameters (dosage, pH, temperature, and settling time with removal parameters (TSS, TD, COD, BOD, and COLR) at a 95% confidence level. Minitab version 17.0 software was used for the statistical analysis. A *p* value > 0.05 and F value < 1.0 implies the effect of each parameter or the data is statistically significant.

### Brownian coagulation–flocculation kinetic theory

**Figure 3:**
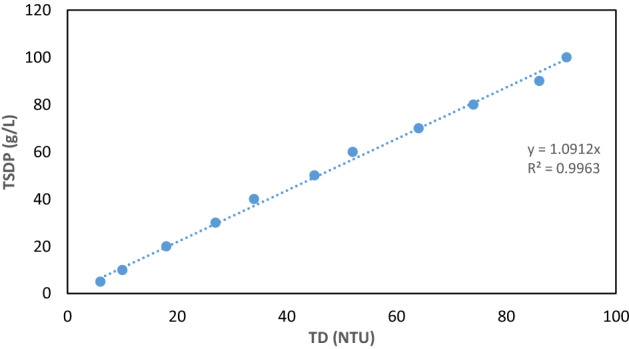
Total dissolved and suspended solids (TDSP) plots for aquaculture effluent (AQEF).

The coagulation data were fitted into Eqs. (2–3), to determine whether the mechanism of the CF treatment of AQEF with PNSC adheres to the von Smoluchowski’s perikinetics concept^52^ using the regression coefficient (R^2^) as the criterion^47^. The coagulation and aggregation kinetics were investigated by plotting 1/C_t_ with time (t) (using Eq. (2)) and Ln C_t_ with t (using Eq. (3))^43,53,54^. TD particle concentration was derived by converting values of TD (in NTU) to particle concentration (TDSP–total dissolved and suspended solids) (in mg L^−1^) using a calibration factor of 1.0912 (Fig. 3).

2 $$\frac{1}{{C_{t} }} - \frac{1}{{C_{0} }} = K_{b} t\quad ({\text{At}}\,\,\alpha = {1})$$

3 $$Ln C_{t} = - K_{b} t + Ln C_{0} \quad ({\text{At}}\,\,\alpha = {2})$$

where *C*_o_ and *C* are the initial TD (in mg L^−1^) and TD (in mg L^−1^) at any given period, *t*; ${K}_{b}$ (the constant of reaction rate); *α* is the reaction order.

*β*_*F*_ is a function of flocculation transport for the mechanisms of shear, Brownian, and differential sedimentation which is described by Eq. 4^52^ given as:

4 $${\beta }_{F}= \frac{8}{3}\frac{{\varepsilon }_{e}{K}_{BC}T}{\eta }$$

where *K*_*BC*_ is 1.38064852 × 10^–23^ J K^−1^ (the Boltzmann’s constant), *η* is 2.6 m Pa s (the viscosity of the AQEF), ${\varepsilon }_{e}$ is the efficiency of collision, and *T* = absolute temperature. The rate of decline in the concentration of AQEF particles ($-{r}_{p}$) at early Brownian kinetic coagulation (i.e. 30 min) is determined through Eq. 5^55,56^:

5 $$-{r}_{p}=-\frac{{dC}_{t}}{dt}={{K}_{b}C}_{t}^{\alpha }$$

where *α* is the coagulation reaction order, *K*_*b*_ is the Menkonu constant rate of coagulation, and *C*_*t*_ is the concentration of the particles (the total suspended and dissolved particles (TDSP) at *t*). ${K}_{b}$ can be obtained through Eq. 7^53,57^:

6 $${K}_{b}= \frac{1}{2}{\beta }_{F}={\varepsilon }_{e }{K}_{sb}$$

7 $${K}_{\mathrm{sb}}=\frac{4{K}_{BC}T}{3\eta }$$

where ${K}_{sb}$ is the von Smoluchowski’s rate constant for fast coagulation. The diffusivity (${D}^{1}$) can be evaluated through Eq. 8^58,59^:

8 $${D}^{1}= \frac{{K}_{sb}}{8\pi r}$$

where *r* is the particle’s radius, *r* and ${B}_{ff}$ (the friction factor) can be evaluated using Eqs. 9 and 10:

9 $$r= \frac{{\beta }_{F}}{6\eta \pi }$$

10 $${B}_{ff}= \frac{{D}^{1}}{T}{K}_{b}$$

The negative values in the rate equation reflect the reduction in TDSP (in mg L^−1^) as time increases. The degree of coagulation was evaluated from values of ${\varepsilon }_{e}$ and ${\tau }_{f1/2}$.

In practice, the particle distribution plot for CF with time may be depicted as follows Eq. 11^60^:

11 $$\frac{{C}_{p }(t)}{{C}_{0}}= \frac{{\left[\frac{1}{{\tau }_{f}}\right]}^{p-1}}{{\left[1+ \frac{t}{{\tau }_{f1/2}}\right]}^{p+1}}$$

where the values for *p* corresponds to the singlets (*p* = 1), doublets (*p* = 2), and triplets (*p* = 3) class of particles; ${\tau }_{f}$ is the fast coagulation period and half-life (${\tau }_{f1/2})$ evaluated using Eqs. (12–13)^59,61–63^:

12 $$\tau_{f} = {\raise0.7ex\hbox{$1$} \!\mathord{\left/ {\vphantom {1 {C_{0} K_{b} }}}\right.\kern-\nulldelimiterspace} \!\lower0.7ex\hbox{${C_{0} K_{b} }$}}$$

13 $$\tau_{f1/2} = {\raise0.7ex\hbox{$1$} \!\mathord{\left/ {\vphantom {1 {0.5C_{0} K_{b} }}}\right.\kern-\nulldelimiterspace} \!\lower0.7ex\hbox{${0.5C_{0} K_{b} }$}}$$

### Cost estimation and energy consumption

Models associated with estimating costs have to be specific, with detailed implementation methodology. Also, models need to analyze cost against important material specifications^64^. In this current work, the total cost (${T}_{c}$) for the treatment of 1 L of the AQEF was evaluated using the expression shown in Eq. (14):

14 $${T}_{c}={C}_{p}+{C}_{L}+{C}_{ce}$$

where ${C}_{L}$ is the cost of coagulant production and ${C}_{ce}$ is the cost of energy.

The energy consumption (*E*) was evaluated using Eq. (15):

15 $$E= {P}_{m}\left(L\times t \times C\right)$$

where *P*_*m*_ is the power consumption by the machine (40 kW), L is a load factor (in a full mode so L = 1), *t* is the time of usage of the machine (0.25 h), and *C* is the energy estimated cost (€0.14/KWh) in Nigeria as at September 9, 2022.

### Coagulation–adsorption kinetics studies

Coagulation phenomena can be modeled theoretically considering as an adsorption-like process^65^. Polyelectrolytes may destabilize materials by a mechanism that combines the effects of charge and adsorption^40^. A polyelectrolyte must be able to eliminate organic COLR to be universally acceptable for use in the treatment of water^40,66^. In this study, the COLR outputs (mg L^−1^) were investigated for the study of the adsorptive constituent of the coagulation–flocculation process, considering the effectiveness of the adsorption technique for the decrease of COLR from the medium. To analyze the sorption kinetics of the treatment process, the nonlinear *pseudo-first-order (PFO)* Eq. 16^67,68^, pseudo-second-order (PSO) Eq. 17 ^69–71^ and Elovich (Chemisorption) Eq. 18^72^ kinetic models were tested.

16 $${q}_{t}= {q}_{e}\left[1-\mathrm{exp}(-{K}_{1}t)\right]$$

17 $${q}_{t}= \frac{{K}_{2}{q}_{e}^{2}t}{1+{K}_{2}{q}_{e}t}$$

18 $${q}_{t}=\left(\frac{1}{\beta }\right)ln\left(1+\propto \beta t\right)$$

where *q*_*t*_ is the amount of adsorbate adsorbed at time *t* (mg g-^1^), *q*_*e*_ is the adsorption capacity in the equilibrium (mg g-^1^), *K*_1_ is the pseudo-first-order rate constant (min^−1^), *K*_2_ is the pseudo-first-order rate constant (g mg^−1^ min^−1^) and *t* is the contact time (min); *α* is a constant related to chemisorption rate and *β* is a constant which depicts the extent of surface coverage.

### Validity of the kinetic models' fittings

To validate the adsorption kinetics models used in the study, in addition to the fixed correlation coefficient (R^2^), the parameters of Marquardt’s percent standard deviation (MPSD), hybrid error function (HYBRID), and sum of the errors squared (ERRSQ) were also evaluated, which can be described as Eqs. (19–21) respectively:

19 $$\mathrm{MPSD}=100\sqrt{\frac{1}{n-p}{{\sum }_{i=1}^{n}\left(\frac{{{\varvec{q}}}_{{\varvec{e}},{\varvec{e}}xp}-{q}_{e,calc}}{{{\varvec{q}}}_{{\varvec{e}},{\varvec{e}}xp}}\right)}_{i}^{2}}$$

20 $$\mathrm{HYBRID}=\frac{100}{n-p}{{\sum }_{i=1}^{n}\left[{\frac{\left({q}_{e,exp}-{q}_{e,calc}\right)}{{q}_{e,exp}}}^{2}\right]}_{i}$$

21 $$\mathrm{ERRSQ}=\sum_{\mathrm{i}=1}^{\mathrm{p}}{(}_{\mathrm{i}}^{2}{\mathrm{q}}_{\mathrm{e},\mathrm{exp}}-{\mathrm{q}}_{\mathrm{e},\mathrm{calc}})$$

where *q*_*e,exp*_ and *q*_*e,calc*_ (mg/g) are the experimental and calculated amounts of color adsorbed, respectively; n is the number of measurements made and *p* is the number of the test elements.

## Results and Discussion

### SEM and FTIR characterization

**Figure 4:**
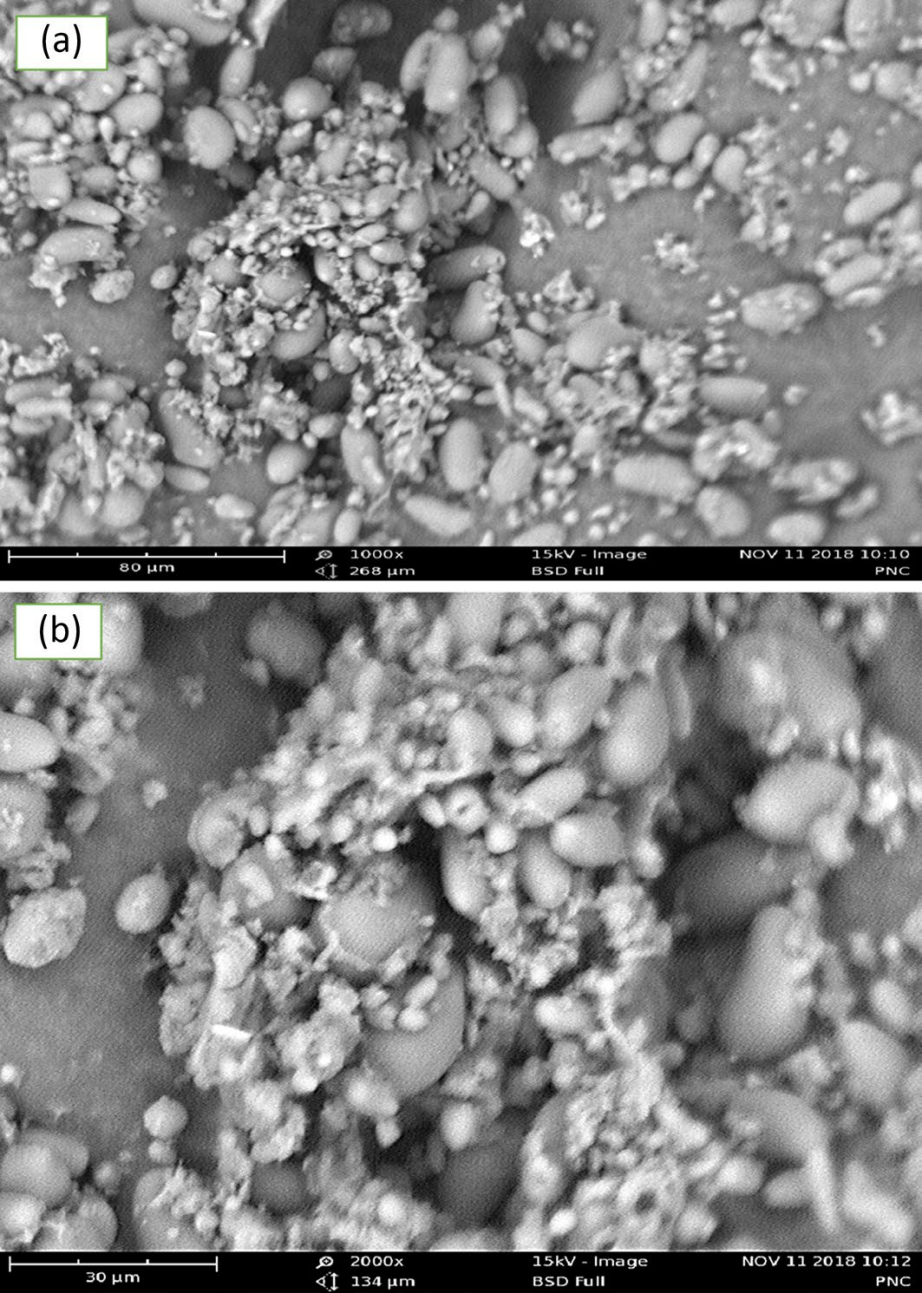
SEM image of *Picralima nitida* seeds coagulant (PNSC) at 1000 × (**a**), and 2000 × (**b**).

The SEM image magnification (1000 × to 2000 ×) of the PNSC is shown in Fig. 4a,b. As seen in Fig. 4, high surface heterogeneity can be observed on the PNSC, suggesting the availability of highly active adsorption sites on the active coagulant for enhanced adsorption mechanisms^73,74^ in the CF process. Also, irregular and rough granular structures can be observed in the PNSC. In particular, these irregular and rough granular surfaces are necessary features of coagulant types concerning the adsorption of dissolved solids and aggregation of suspended solids. These features will promote colloidal particles’ attraction, agglomeration, capturing^75^ and promote their sedimentation.

**Figure 5:**
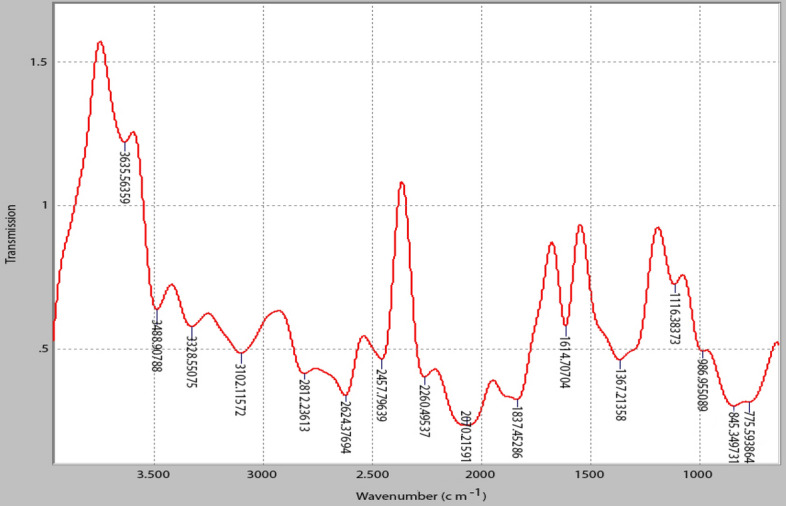
FTIR spectra on active PNSC.

The FTIR spectrums of the PNSC are shown in Fig. 5. The presence of N–H stretching in the spectrum of the PNSC confirmed the existence of amino compounds (protein). The FTIR analysis indicates that the carboxyl (C=O), hydroxyl (O–H), and amino or amide (N–H) groups, in addition to hydrogen bonding, were present in the structure of PNSC. These were proven functional groups active in the CF process^76^. The presence of C=O groups will serve as an anion bridge for divalent metal cations such as Mg^2+^ and Ca^2+^ at the surface of the particle to induce coagulation activity^77^. The O–H stretch, and free hydroxyl of alcohols and phenols, are strong evidence indicated by the sharp band observed on the PNSC. This hydrogen bonding aids particle adsorption. The presence of –OH stretching indicates that the active PNSC samples are hygroscopic in nature^78^.

### Effect of process factors

#### Effect of PNSC dosage on the treatment of AQEF

**Figure 6:**
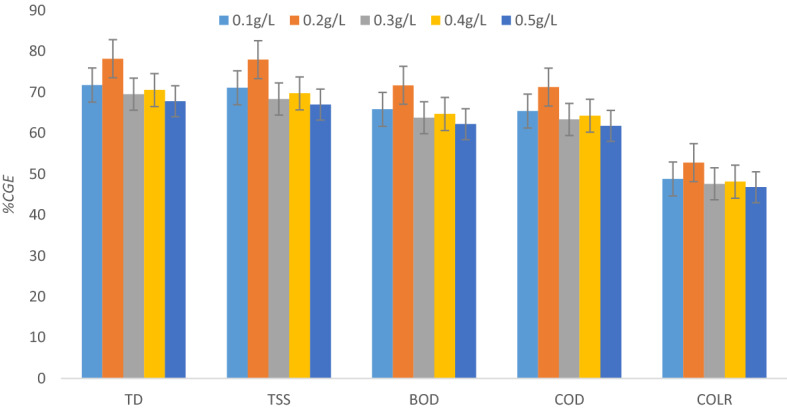
Effect of PNSC dose on pollutants elimination on AQEF at pH 7.9 and 60 min.

The optimization of coagulant/flocculant dosages provides an understanding of the scientific principles behind the coagulation–flocculation processes. The charge neutralization and adsorption mechanism for the removal of contaminants can be achieved with the optimum dosage of the bio-coagulant being provided. Figure 6 depicts the antagonistic impact of PNSC dosage on TSS, TDS, BOD, COD, and COLR removals from AQEF at pH 7.9 for 60 min. The findings indicated that 0.2 g L^−1^of PNSC was sufficient for obtaining the highest coagulation removal efficiencies (% CGE)—TD, TSS, BOD, COD, and COLR reductions, which transcends to 78.22, 77.99, 71.73, 71.28, and 52.80% on AQEF. The PNSC’s performance recorded its worst when experimented with the dosage of 0.5 g L^−1^which produced maximum TD and TSS removal efficiencies ≤ 67%. The summary of the result obtained would be indicative of 0.2 g L^−1^ of PNSC as an ideal dose for treatment of AQEF. The performance output indicates that introducing a dosage of PNSC higher than the optimum (0.2 g L^−1^) caused the particles’ surface charges to reverse due to lots of adsorption sites per PNSC particle, which inhibited the effectiveness of removal and inter-particle bridging^79,80^.The influence of the PNSC-induced flocculation process was observed to be linked to the optimal dosage and subsequent efficiency of removal^81,82^.The findings suggest that the optimum dose (0.2 g L^−1^) of the active PNSC was most effective for the clarification of TD and other contaminants present in the aquaculture effluent. The low optimum dosage of PNSC ≤ 0.2 g L^−1^ indicates that the main mechanisms behind the CF process are charge neutralization and adsorption^82,83^. Also, the lower dose of 0.2 g L^−1^ being optimal will minimize sludge generation while reducing expense and environmental impact^84^. The one-way ANOVA test was performed to understand the significance of the changes in dosage with removal parameters (TSS, TD, COD, BOD, and COLR) at a 95% confidence level. A *p* value of 0.568 was evaluated at ($p>0.05$). An *F* value (0.75) < 1 recorded implies the effect of dosage on the reduction of TSS, TD, COD, BOD and COLR from AQEF using PNSC is not significant statistically.

#### Impact of pH on the treatment of AQEF

**Figure 7:**
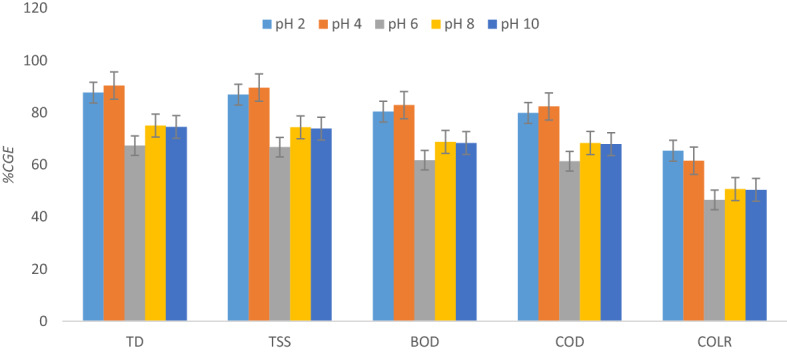
Effect of AQEF pH on pollutants elimination at 60 min and 0.2 g L^−1^.

The outline of the Fig. 7 shows the effect of pH modification over a range of 2 to 10 at a settling time of 60 min and under the influence of an optimum PNSC dosage of 0.2 g L^−1^on the reduction of TD, TSS, BOD, COD, and COLR in AQEF. The findings proved that the maximal TD, TSS, BOD, COD, and COLR removal rates correspond to 90.35, 89.57, 82.83, 82.34, and 65.37%, respectively. The outcome established that the optimal reduction of the selected contaminants was significantly successful in acidic media. Moreover, it can be observed from the plot of Fig. 7 that the PNSC performed poorly at pH 6 which plots removal efficiency ≤ 65%. The authors reasoned that the impact of increasing pH > 4 on particle electrophoretic mobility resulted in little surface charge neutralization, thus resulting in low contaminant removal rates. The pH modification above neutral (pH 7) transcends to the occurrence of comparable charge with more availability of OH–, leading to a rise in electrostatic repulsion of the AQEF particles. The interpretation of Fig. 7 suggests that operating PNSC outside the optimum pH window resulted in poor stability of the suspended particles in the AQEF^40,64^. Furthermore, it can be inferred from the results that particle agglomeration was enhanced at low pH due to decreased in inter particle repulsions^85,86^ and changing effluent chemistry. The outcome of the PNSC-driven pH modification of AQEF is an indication that charge neutralization and inter-particle bridging effect of the polymeric material aided adsorption, and agglomeration of the various contaminants under investigation, thereby converting the particles to flocs that settle easily. Also, a combination of the carbohydrate content (usually the C=O and –OH groups), and the active metals from the complex salt extraction solution (MgCl_2_ + NaCl + KCl + CaCl_2_) was deposited on the surface of the active PNSC allays the fear associated with the protein denature at optimum pH 4. This outcome accounted for the reduction of TD, TSS, BOD, COD, and COLR concentrations which transcends to removal efficiencies ≥ 85%. The result also confirmed the PNSC-driven coagulation–flocculation treatment favored the removal of TD and TSS compared to BOD, COD, and COLR present in AQEF under acidic media with a corresponding removal efficiency ≥ 90%. In a study conducted by Beltrán-Heredia et al.^87^**,** the proteinic—cationic characteristics of *M. oleifera*, according to the authors, may indicate improvement of the coagulant activity at a low pH of 4 where the efficiency was decreased from pH 4–10; they observed a similar result for a tannin-based coagulant were a swift decrease was seen. The optimum pH (4) reported for PNSC seems to support other findings reported about green biocoagulants in published works of Menkiti and Ejimofor^62^, Ejimofor et al.^88^, and Okolo et al.^89^. Considering the increase in PNSC-driven TD and TSS removal efficiency, the authors reasoned that the pH window had a significant antagonistic effect on the sorption capacity of PNSC compliance with AQEF. The finding is a reasonable agreement reported in published works in the literature^62,88,89^. The authors reasoned that PNSC like most chemicals (alum and ferric-based coagulants), is probably acidic in nature which produces a drop in pH in the water medium. The pH adjustment of the AQEF resulted from the changing effluent chemistry. The optimal pH (4) indicates that microbial and bacterial activity in the finished effluent will be reduced and is considered an advantage in this case. A *p* value of 0.013 ($p<0.05$) and *F *value = 4.19 > 1 were evaluated. This implies the effect of pH on the reduction of TSS, TD, COD, BOD and COLR from AQEF using PNSC is statistically significant.

#### Effect of temperature on TD, TSS, BOD, COD, and COLR reduction

**Figure 8:**
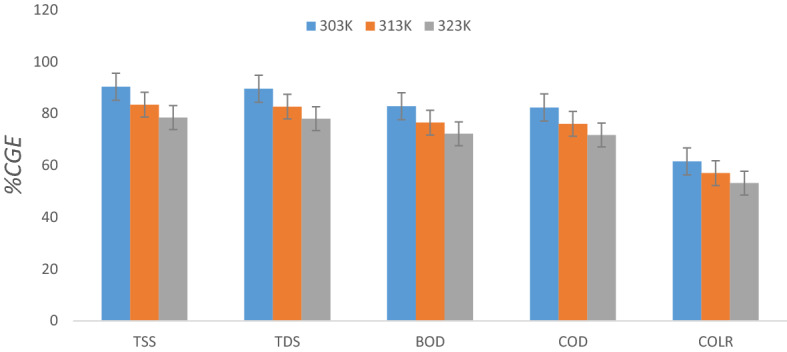
Effect of temperature on pollutants reduction at settling 60 min, 0.2 g L^−1^, and pH 4.

Figure 8 was drawn to determine the optimum temperature for the coagulation–flocculation process. The outline of Fig. 8 shows the impact of temperature on TD, TSS, BOD, COD, and COLR reductions on AQEF compliance with PNSC. The analysis of the effect of temperature on the clarification efficacy of the active coagulant was tested at 303, 313, and 323 K under optimal operating conditions (dosage of 0.2 g L^−1^ and pH). The outcome confirmed that at 303 K, maximum TD, TSS, BOD, COD, and COLR elimination rates corresponding to 90.35, 89.57, 82.83, 82.34, and 61.53% were attained. The output corresponds to the height of the bar charts presented in Fig. 8. The findings established that the least TD, TSS, BOD, COD, and COLR removal rate was recorded operating the PNSC-driven CF treatment at the temperature of 323 K. The removal rate of the contaminants contained in the AQEF decreased intermittently as the temperature increased. The results obtained confirmed that the optimum temperature that produced the best removal rate was recorded at 303 K. The author reasoned that the decrease in TD, TSS, BOD, COD, and COLR removal rate with rising temperature can be attributed to the haphazard movement of pollutant particles produced by increased kinetic energy. The increase in temperature prevented the particle’s trapping to the PNSC surface from forming flocs and led to a reduction in floc size^90^. The colloidal particles formed from the CF process spread widely apart rather than agglomerating together to create bigger flocs and prevent the particles from settling faster^85,91^.The floc strength ultimately deteriorated, leading to breakage^39^. Consequently, fewer large flocs were developed. The finding suggest viscosity of AQEF was altered due to temperature rises, thus decreasing the removal efficiency^92^.

Most feasibly, increasing temperature above the optimum (303 K) would impair the bio-coagulant performance along with the adsorption potential of its active sites and functional groups. These findings are consistent with the observation reported from the influence of the optimum dosage, and pH on PNSC-driven coagulation treatment of the AQEF on TSS, and organics removal from AQEF. A *p* value of 0.001 ($p<0.05$) and *F* value = 12.34 > 1 were evaluated; this outcome implies that the effect of temperature on the reduction of TSS, TD, COD, BOD and COLR from AQEF using PNSC is significant.

#### Settling time influence on TD, TSS, COD, BOD, and COLR reduction

**Figure 9:**
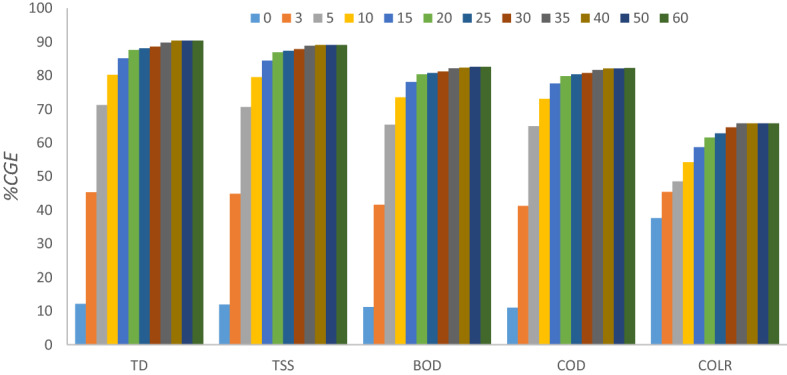
Effect of settling time on TD, TSS, BOD, COD, and COLR elimination on AQEF at 0.2 g L^−1^, pH 4, and 303 K.

Figure 9 illustrates the influence of settling time on contaminant removal from AQEF compliance with PNSC. The impact of the coagulation–flocculation settling period on the reduction of the pollutants (TD, TSS, BOD, COD, and COLR) was investigated by varying the settling time from 0 to 60 min. The result showed that pollutants elimination improved substantially as settling time increased until equilibrium was reached at optimum operating conditions (pH 4, a dosage of 0.2 ml^−1^, and 303 K). The result shows that, across all contaminants, the removal efficiency increased consistently from 10 to 92% as settling time increased from 0 to 60 min until stability was attained. The authors reasoned that the rapid settling time was aided by the formation of larger and denser flocs resulting from the biopolymer chain’s adhesion to the particles in the effluent and the charge on the surface of the active coagulant^39^. Across all contaminants removed from the effluent, equilibrium was attained after 40 min. This outcome proved that the coagulant aligns with pollutants in the effluent, leading to the reduction of the contaminants in the AQEF. At the optimal dose (0.2 g L^−1^) and equilibrium settling time (40 min). The maximum TD, BOD, and COD removal corresponding to 90.35, 82.38, and 82.11% were realized. While removal rate of 65.77%, and 88.84% were recorded for COLR and TSS at 35 min. The performance output was consistent with the observations reported on green coagulants by several authors^46,93^. The experimentation indicates that settling time, pH, and the temperature had the most significant effect on the overall performance of the active PNSC as a green coagulant. The removal efficiency of PNSC was largely dependent on the antagonistic effect of pH, temperature, and settling time. The dosage had a ceiling effect on the clarification efficacy of the bio-coagulant, with a low tendency to form sludge^94^. Also, a *p* value of 0.001 ($p<0.05$) and *F* value = 24.23 > 1 were evaluated. This output confirmed the effect of settling time on the reduction of TSS, TD, COD, BOD, and COLR from AQEF using PNSC is significant statistically.

### Brownian coagulation–flocculation (CF) kinetics on the process

**Figure 10:**
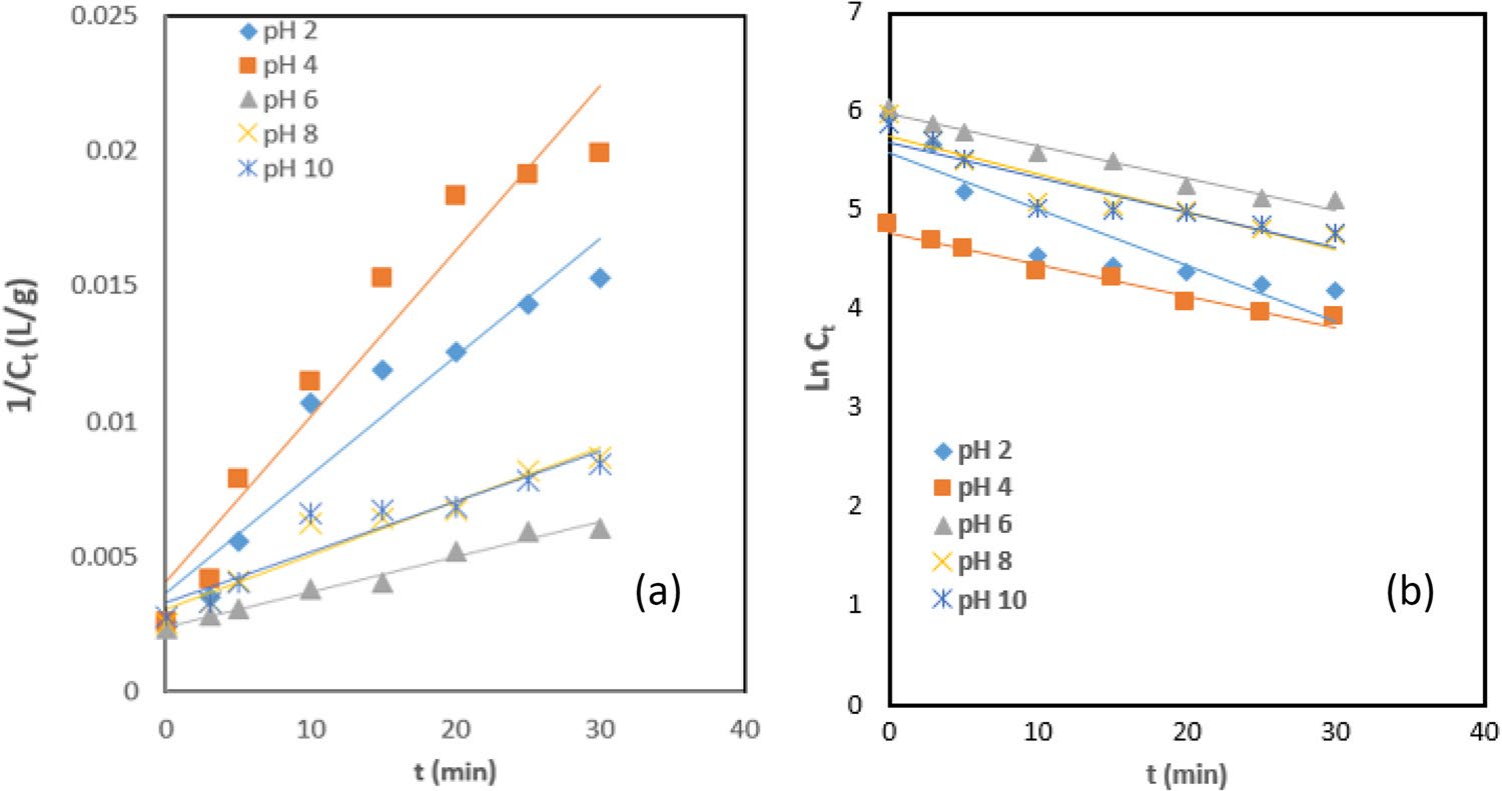
(**a**) First-order and (**b**) Second-order CF kinetics at 0.2 g PNSC L^−1^ and 303 K.

The values of *K*_*b*_ and C_0_ were obtained from Fig. 10a,b. Figures 10a, b show the first and second-order CF kinetics at the optimum operating conditions (0.2 g PNSC L^−1^ and 303 K), obtained by comparing Eqs. 2 and 3, respectively. The summary of the kinetic parameters recorded at the optimum conditions are shown in Tables 2 and 3. The kinetics investigations proved that the PNSC-driven coagulation rate constant increased intermittently from2.4 × 10^–3^ L mg min^−1^ to 4.1 × 10^–3^ L mg min^−1^ with a range of coefficient of determination 0.9333 ≤ R^2^ ≤ 0.9796 for first-order (Table 2). The maximum rate constant (4.1 × 10^–3^ L mg min^−1^) was recorded at pH 4 and transcends to optimum TD and TSS removal efficiency ≥ 90%. A similar outcome was observed with the second-order CF kinetics (Table 3), with the maximum value of the rate constant (6.35 × 10^–2^ L mg min^−1^) with a corresponding R^2^ (0.9679) recorded at pH of 4. The least flocculation rate recorded at pH 6 translates to contaminant reduction efficiency ≤ 75%. The kinetic data confirmed that, at the initial stage, the PNSC-driven coagulation dynamics were synergetic with the first-order and second-order kinetic models (where α = 1 and 2). The authors reasoned that the initial stages where the CF dynamics obeyed 1st-order kinetic is attributed to a shift from theoretical expectation but in line with empirical evidence^95,96^. As the coagulation reaction proceeded towards the optimum the dynamics of the PNSC in AQEF adjusted to the 2nd-order (perikinetics flocculation) with a corresponding R^2^ ≤ 0.9796. Consequently, the summary of the kinetic parameters (Table 3) was estimated for the second-order PNSC-driven CF kinetic parameters.

**Table 2:** CF kinetics at 0.2 g PNSC L^−1^ and 303 K at α = 1.

Factor	pH 2	pH 4	pH 6	pH 8	pH 10
R^2^	0.9101	0.9333	0.9796	0.9361	0.8951
*K*_*b*_ (L mg^−1^ min^−1^)	0.0037	0.0041	0.0024	0.0031	0.0033
C_o_ (mg L^−1^)	2500	1666.7	10,000	5000	5000
*r*_*p*_ (mg min^−1^)	− 0.0037C_t_	− 0.0041C_t_	− 0.0024C_t_	− 0.0031C_t_	− 0.0033C_t_

**Table 3:** CF kinetic parameters at 0.2 g PNSC L^−1^ and 303 K at α = 2.

Factor	pH 2	pH 4	pH 6	pH 8	pH 10
R^2^	0.9292	0.9679	0.9931	0.9313	0.9457
*K*_*b*_ (L mg^−1^ min^−1^)	0.0568	0.0635	0.0322	0.038	0.0033
C_o_ (mg L^−1^)	267.66	119.26	392.05	313.34	298.211
*r*_*p*_ (mg min^−1^)	− 0.0568*C*_*t*_^2^	− 0.0319*C*_*t*_^2^	− 0.0322*C*_*t*_^2^	− 0.038*C*_*t*_^2^	− 0.0033*C*_*t*_^2^
${\tau }_{f1/2}$ (min)	3.801	1.893	3.156	2.977	2.647
${\tau }_{f}$ (min)	7.6014	3.786	6.3121	5.9535	5.2932
*Β*_*F*_ (L mg^−1^ min^−1^)	0.1136	0.127	0.0644	0.076	0.071
*K*_*sb*_ (L min^−1^)	2.14 × 10^–21^	2.14 × 10^–21^	2.14 × 10^–21^	2.14 × 10^–21^	2.14 × 10^–21^
${\varepsilon }_{e}$ (L mg^−1^)	2.65 × 10^19^	2.96 × 10^19^	1.52 × 10^19^	1.77 × 10^19^	1.65 × 10^19^
*D*^*1*^	3.68 × 10^–20^	3.29 × 10^–20^	6.5 × 10^–20^	5.5 × 10^–20^	5.89 × 10^–20^

The findings from the kinetic results established that the highest collision efficiency ${(\varepsilon }_{e}$) of 2.96 × 10^19^ L mg^−1^ was recorded at pH 4. The performance of PNSC in AQEF proved that higher values of ${\varepsilon }_{e}$ resulted in high energy of kinetics, indicating a tendency to lower the zeta potential. The performance output of PNSC in AQEF is consistent with the pH results reported for green coagulants in published research works available in the literature^97,98^. The value of the half-life ${(\tau }_{f1/2 })$ for the CF treatment is an important parameter that is linked to optimal particle aggregation^99^. The values of half-life (${\tau }_{f1/2 })$ obtained from the PNSC-driven kinetics decreased intermittently as the pH of AQEF increased from 4 to 6. The outcome leads to colloidal destabilization^100^ at ${\tau }_{f\frac{1}{2}}\le 1.80$ min, yielded optimum coag-flocculation efficiency ≥ 90.20%. The low values of ${\tau }_{f1/2}$ recorded confirmed the theory of fast coagulation is prevalent on CF treatment of the AQEF^101^. The values ofthe PNSC-driven flocculation period (${\tau }_{f })$ and half-life (${\tau }_{f1/2 })$ is shown in Table 3. The maximum value of Brownian collision factor (*Β*_*f*_) ≥ 0.13 was recorded at pH 4.This output is connected to the collision efficiency^96,102^ necessary to reduce the double layer compression or destabilize the particles to achieve low ${\tau }_{f1/2}$ values necessary for rapid flocculation to occur.The optimum performance of PNSC in AQEF was prevalent on the minimum particle concentration (C_0_ = 119.26 mg L^−1^) at flocculation period (t_*f*_ = 3.78 min) and half-life (${t}_\frac{1}{2}\hspace{0.17em}$= 1.90 min). These outputs were recorded at R^2^ ≤ 1, confirming statistical fit of the CF data to the kinetic model were significant. The findings established that the best performance of the biocoagulant corresponds to the maximum coagulation–flocculation rate constant (*K*_*b*_) ≥ 0.06 at pH 4 (Table 3), which transcend to TD, TSS, BOD, COD, and COLR efficiencies of removal corresponding to 90.35, 89.57, 82.83, 82.34, and 65.37%, respectively.

### Particle distribution behavior of the process

The time-evolution and aggregating of the different classes of particles: singlets, doublets, and triplets based on size variation were employed to forecast their behavior as time changes. The time-evolution and particle aggregation parameters presented in Table 4 were evaluated following Eq. 11. The distribution pattern of the different aggregates formed in terms of the particle concentration per cubic meter in AQEF was investigated using values of ${\tau }_{f1/2}$, C_0_ and *K*_*b*_ derived from 2^nd^ order kinetics at optimum conditions (Table 3). The time-evolution and particle distribution (Table 4) was drawn to illustrate the computed values of aggregates (C_1_-ƩC) of the triplet, doublet, and singlet particle counts, as well as the overall particle counts.

**Table 4:** Particle evolution with time for the AQEF/PNSC process at *K*_*b*_ = 0.0635 Lg^−1^ min^−1^, *C*_0_ = 119.26 gL^−1^and $\tau$= 1.90 min.

Time (s)	C1	C2	C3	ƩC
0	208.3333	0	0	208.3333
180	17.0068	1.644935	0.10408	18.75582
300	10.54852	0.592503	0.025674	11.1667
600	5.411255	0.148145	0.003556	5.562957
900	3.63901	0.065843	0.001091	3.705944
1200	2.741228	0.037037	0.000468	2.778733
1500	2.198769	0.023704	0.000242	2.222715
1800	1.835536	0.016461	0.000141	1.852138
2100	1.575299	0.012094	8.94E-05	1.587482
2400	1.379691	0.009259	6.01E-05	1.38901
3000	1.105217	0.005926	3.09E-05	1.111173
3600	0.921829	0.004115	1.8E-05	0.925962

**Figure 11:**
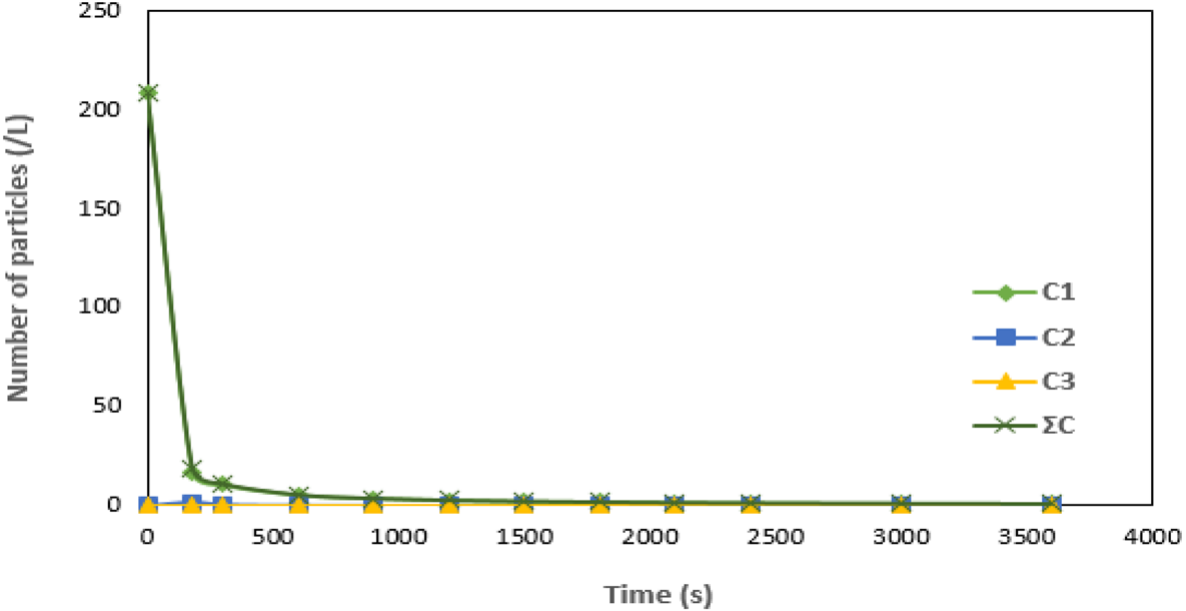
Particle distribution graph for PNSC on AQEF at ${\tau }_{f1/2}\hspace{0.17em}$= 1.90 min, *C*_0_ = 119.26 g L^−1^ and *K*_*b*_ = 0.0635 Lg^−1^ min^−1^.

Figure 11 was drawn to illustrate the time evolution and particle distribution for the impact of PNSC on AQEF. The summary of the distribution of the concentrations of the particles per cubic meter is presented in Table 5. The varying particles concentration number (C1-ƩC) with time (t) at the optimum operating conditions were evaluated by substituting values of ${\tau }_{f1/2}$=1.90 min, *C*_0_ = 119.26 g L^−1^, and *K*_*b*_ = 0.0635 Lg^−1^ min^−1^, into Eq. 11. Figure 11 shows the time evolution and aggregation of the particles that characterize the AQEF. The plot depicts the trajectory of the agglomeration of the particles and the settling characteristics of the various aggregates (C1-ƩC) with time (t). The rapid destabilization of singlets accompanied the formation of doublet and triplet counts. The singlet class particles declined more rapidly than the overall number of particles^93,103^.The authors reasoned that Brownian coagulation dominated the fundamental particles^104^. The mechanism of aggregation of the particles was described by a combination of charge neutralization and sweep flocculation^11^. The curvatures of the curves in Fig. 11 show that the estimate of singlet, doublet, and triplet aggregates dropped systematically throughout time. The protonated amine groups often destabilize the negative charges and the zeta potential, lowering or eliminating the DLVO energy barrier and allowing for more species interactions^98^. Conclusively, it can be inferred from the outcome of the time evolution and particles aggregation that charge neutralization mechanism occurred under the influence of the optimum dosage (0.2 g L^−1^), and most of the pollutant particulates were cleaned up from the effluent medium via gravity, settling after being entwined in the protein complex.

**Table 5:** PNSC-driven adsorption kinetic parameters for COLR removal on AQEF optimum conditions.

**PFO**	
*K*_1_ (min^−1^)	0.1143
*q*_*e*_ (mg/g)	0.2509
*h*_0_(mg/g/min)	0.0287
R^2^	0.9979
ERRSQ	0.00002
HYBRID	0.0011
MPSD	7.2838
**PSO**	
*K*_2_ (g/mg/min)	0.7847
*q*_*e*_ (mg/g)	0.2560
*h*_0_(mg/g/min)	0.0514
R^2^	0.9905
ERRSQ	0.0000822
HYBRID	0.0109
MPSD	15.5148
**Chemisorption**	
*B*	7.7963
*α* (mg/g.min)	0.0358
R^2^	0.9737
ERRSQ	0.0001
HYBRID	0.0203
MPSD	25.7411

### Coagulation–adsorption kinetics and validity results

**Figure 12:**
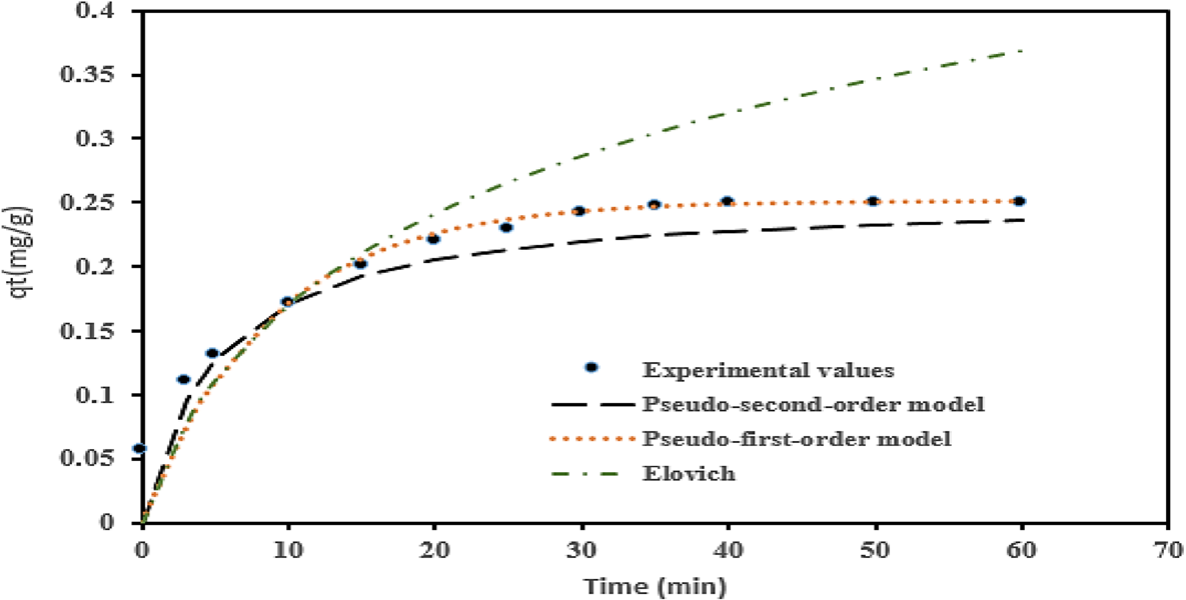
Kinetics plots for COLR removal on AQEF at pH4 and 0.2 g PNSC L^−1^.

The nonlinear Lagergren pseudo-first-order, pseudo-second-order, and Elovich (chemisorption) kinetic models (Eqs. 16–18) were tested on the data and used to describe the mechanism of the adsorptive uptake of COLR from AQEF using PNSC at optimum conditions. Figure 12 illustrates the sorption kinetics plots obtain from values of *q*_*t*_ plotted against time. The summary of the adsorption kinetics parametric values is presented in Table 5. The result shows that under the optimum conditions, the range of values of the correlation coefficient 0.9737 ≤ R^2^ ≤ 0.9979, and values of estimated error-squared in the range of 0.0001 ≤ ERRSQ ≤ 0.00002 were recorded for the coagulation–adsorption kinetics. The highest adjusted-R^2^ ≤ 1.0 indicates that a particular model best fits the adsorption kinetic data^108^. The model with the lowest values of Marquardt’s percent standard deviation (MPSD), hybrid error function (HYBRID), and the errors squared (ERRSQ) value best suits the data to the kinetic model. The use of error functions is statistically more accepted than the adjusted R^2^ since it indicates low error obtained for the data. The findings from the adsorption kinetics established that the lowest model statistical metrics: HYBRID (0.0011), ERRSQ value (2 × 10^–5^), and MPSD (7.28), were recorded for the PFO kinetic, as shown in Table 5. The results showed that the PFO kinetic model best describes the coagulation–adsorption mechanism. The outcome indicated that PNSC-driven adsorptive uptake of COLR from AQEF conformed to the PFO model. This outcome suggests that the process of COLR reduction from AQEF is not chemically controlled. The models’ adjusted-R^2^ (0.9979) was closest to unity, confirming the goodness of fit of the kinetic data^94^. The maximum sorption capacity (*q*_*e*_ = 0.2509 mg/g) recorded for the PFO model correspond to 70% COLR removal efficiency.

### Comparative analysis of PNSC with other coagulants

The comparative analysis of PNSC and other green coagulants on AQEF expressed in terms of %TD, %TSS, %COLR, %BOD, and %COD measurements are presented in Table 6. The comparative analysis (Table 6) shows a consistency in the overall performance of PNSC with the published results reported by other researchers. The findings from the current research work established efficacy of PNSC is as a natural coagulant is favorable for clarification of TD from AQEF with a higher removal rate (90.23%) compared to 89% reported for *Moringa oleifera* seed extract^105^, 88% for Brachyura shell waste^106^, 88% TD removal efficiency reported for bio-polymer chitosan^107^ applied in the treatment of AQEF. However, the efficacy of PNSC in the removal of TSS (89.90%) was consistently higher than 78.82% reported for *Serratia marcesens*^108^, 62% for bio-polymer chitosan^107^, and 82.70% for Neem leaves^109^ in AQEF. The COLR reduction rate of 65.70% was obviously higher than 54.77% reported for *Parkia biglobosa* seeds^10^ in the AQEF system. The COD and BOD removal efficiency of 82.34 and 82.11% recorded for PNSC in the AQEF system was also reported higher than the 75% reported for *Sesamum indica*^12^, 62% and 52% efficiencies of removal reported for bi-polymer chitosan in AQEF system^107^. The authors reasoned that the circumstances under which these efficiencies were achieved are proven from the time evolution and aggregate distribution, coagulation, and adsorption kinetics. Generally, most writers focused primarily on the decrease of TD and TSS rather than on the organic components (BOD and COD) and colour removal, which was reported by the current study. The study demonstrated that the primary substances eliminated by the PNSC-driven coagulation–flocculation treatment of AQEF are turbidity and suspended particles. Consequently, instead of using chemical-based substances, natural coagulants such as PNSC can be employed.

**Table 6:** Comparative analysis of PNSC with other coagulant sources on AQEF.

Contaminant investigated	Conditions	Results (%)	References
TD	pH = 6, coagulant type: Brachyura shell waste extract, dosage: 1.8 g/L	88.00	^106^
COLR		91.00	
TD	pH = 6.8, coagulant type: *Serratia marcescens*culture, dosage: 10% v/v	83.95	^108^
TSS		78.82	
TD	pH = 7.28, coagulant type: *Moringa oleifera* seeds extract, dosage: 6 ml	89.00	^105^
BOD		100	
TD	pH = 7.2, coagulant type: Biopolymer chitosan, dosage: 12 mg/L	87.70	^107^
TSS		62.60	
BOD		52.30	
COD		62.80	
NH3		91.80	
Phosphate		99.10	
Bacteria		100.00	
TSS	pH = 7.5, coagulant type: Neem leaves extract, dosage: 0.3 mg/L	82.70	^109^
COLR		81.37 65.80	
TD	pH = 2, coagulant type: *Sesamum indicum* seeds extract, dosage: 0.4 g/L	83.88	^12^
TDS		82.00	
BOD		70.03	
COD		75.99	
TD	pH = 2, coagulant type: *Parkia biglobosa* seeds extract, dosage: 0.3 g/L	80.44	^10^
Salinity		79.98	
COLR		54.47	
TD	pH: 4, coagulant type: *Picralima nitida* seeds, dosage: 0.2 g PNSC/L	90.35	This work
TSS		88.84	
BOD		82.38	
COD		82.11	
COLR		65.77	

### Cost-energy considerations on the treatment process

Within a given environmental terrain where there are product concepts, alongside quality (and environmental) data, there is the cost data that has to be fed into the concept comparison matrix. This above-mentioned perspective was among the green quality function that Dong et al.^110^ deployed in ascertaining key aspects of the cost estimation for environmentally conscious product development. More so, cost estimation practices continue to evolve over time, fundamentally aiming to increase by accuracy and effectiveness, largely dependent on data appropriateness as well as legitimacy, despite the fact that there is no complete theoretical model^64^. Further, besides exploring cost analysis as a decision-making tool to test for the feasibility of the active coagulant^11^, the cost of the CF operation has to be based on the efficacy of the active coagulant, energy consumption, and technology required to remove contaminants^43^. In this current work, the cost of treating 1 L of AQEF was calculated by taking into account the cost of the following: (a) preparing 0.2 g L^−1^ (optimal dose) of the PNSC; (b) energy, as well as (c) labor. To provide further explanation to these, the preparation of 0.2 g L^−1^ of PNSC would cost €0.17. However, the labor cost would be projected at €3.24, whereas the energy cost would be projected at €0.14/KWh. With these, computing the total cost appeared feasible, and generated €4.81, bearing in mind the considerations of costs of 0.2 g L^−1^ PNSC preparation, labor, and energy. Considering the above results, the techno-economic feasibility of wastewater and effluent handling would most likely necessitate the use of low-cost materials.

## Conclusion

The aquaculture effluent pollutant removal using *Picralima nitida* seeds extract via coagulation–flocculation treatment was investigated. In the current research work, pilot scale experimentation conducted, and the results was focused on the bio-coagulation performance, coagulation–flocculation/adsorption kinetics, particle temporal evolution, and the cost–benefit analysis of employing PNSC to treat 1 L of AQEF. The results obtained show that TSS, TD, COD, BOD, and COLR concentration in aquaculture effluent (AQEF) were reduced using the novel PNSC. The effects of PNSC dosage, pH, temperature, and settling time on the reduction of pollutants were examined and their statistical significance were tested via ANOVA. The data were explored through the sorption and flocculation kinetics equations. The PNSC possessed the amino (N–H) and hydroxyl (O–H) groups– proven to be very beneficial for coagulation–flocculation. The efficacy of the active coagulant in the aquaculture system translates to the order of removal of the pollutants TD > TSS > organics (BOD and COD) > COLR. Maximal TD reduction = 90.35%, TSS = 88.84%, COD = 82.11%, BOD = 82.38% and COLR = 65.77% at 0.2 g L^−1^dosage of PNSC, pH 4, and 303 K was achieved. The *p* values and F-values obtained from ANOVA analysis inferred that the pH, temperature and settling time had a significant effect on the pollutants removal. Von Smoluchowski’s kinetics fit the results. At perikinetics condition, the *K*_*b*_ (reaction rate) and ${t}_{f\frac{1}{2}}$ (half-life) correspond to 0.0635 L g^−1^ min^−1^ and 1.9 min under the ideal circumstances. The sorption data fitted the Lagergren more than the Ho adsorption model. The total cost of using PNSC to handle 1 L of AQEF was €4.81. The final BI (0.98 > 0.3) suggests that the pretreatment using the CF via PNSC improved the biodegradability of AQEF. The PNSC-driven coagulation process demonstrates a feasible solution for the clarification of TD, TSS, COD, COLR, and BOD removal from the aquaculture effluent system. Although an optimum pH (4) recorded vary slightly outside EPA standard for effluent discharge (5.5 ≤ pH ≤ 9), the outcome calls for further studies to be conducted to ascertain for the pH modification of PNSC in effluent system. Under such prevalence, PNSC can be classified as a flocculant or coagulant aid. Other challenges include experimental scale-up for industrial acceptance of PNSC and lack of research regarding the practical usage of PNSC with other effluent sources and coagulant aid. To overcome these shortcomings, the direction of future research could focus on undertaking modeling simulations to estimate the potential scale-up feasibility considering specifically contexts of different locations, testing PNSC with coagulant aids where pH modification is concerned, considering the availability of materials (to make PNSC), as a start.

## Data Availability

The datasets used and/or analyzed during the current study are available from the corresponding author upon reasonable request.

## References

[1] Turcios AE, Papenbrock J (2014). Sustainable treatment of aquaculture effluents—What can we learn from the past for the future?. Sustainability.

[2] Burford MA, Costanzo S, Dennison W, Jackson C, Jones A, McKinnon A, Preston N, Trott L (2003). A synthesis of dominant ecological processes in intensive shrimp ponds and adjacent coastal environments in NE Australia. Mar. Pollut. Bull..

[3] Andreotti V, Chindris A, Brundu G, Vallainc D, Francavilla M, García J (2017). Bioremediation of aquaculture wastewater from *Mugil cephalus* (Linnaeus, 1758) with different microalgae species. Chem. Ecol..

[4] Soletto D, Binaghi L, Lodi A, Carvalho J, Converti A (2005). Batch and fed-batch cultivations of Spirulina platensis using ammonium sulphate and urea as nitrogen sources. Aquaculture.

[5] Snow A, Ghaly A (2008). A comparative study of the purification of aquaculture wastewater using water hyacinth, water lettuce and parrot’s feather. Am. J. Appl. Sci.

[6] Oladoja N, Adelagun R, Ahmad A, Ololade I (2015). Phosphorus recovery from aquaculture wastewater using thermally treated gastropod shell. Process Saf. Environ. Prot..

[7] Darwin D, Sarbaini S, Purwanto S, Dhiauddin F, Ilham M, Fazil A (2017). Wastewater treatment for African catfish (*Clarias gariepinus*) culture by using anaerobic process. Agritech.

[8] Omitoyin B, Ajani E, Okeleye O, Akpoilih B, Ogunjobi A (2017). Biological treatments of fish farm effluent and its reuse in the culture of nile tilapia (*Oreochromis niloticus*). J. Aquac. Res. Dev..

[9] Kim K, Hur JW, Kim S, Jung J-Y, Han H-S (2020). Biological wastewater treatment: Comparison of heterotrophs (BFT) with autotrophs (ABFT) in aquaculture systems. Biores. Technol..

[10] Igwegbe CA, Ighalo JO, Onukwuli OD, Ahmadi S (2021). Bio-coagulation–flocculation of land-based saline aquaculture effluent using parkia biglobosa seeds: turbidity, salinity, and colour reduction. Removal of Pollutants from Saline Water.

[11] Igwegbe CA, Ighalo JO, Onukwuli OD, Obiora-Okafo IA, Anastopoulos I (2021). Coagulation–flocculation of aquaculture wastewater using green coagulant from Garcinia kola seeds: Parametric studies, kinetic modelling and cost analysis. Sustainability.

[12] Igwegbe CA, Onukwuli OD (2019). Removal of total dissolved solids (TDS) from aquaculture wastewater by coagulation–flocculation process using Sesamum indicum extract: Effect of operating parameters and coagulation–flocculation kinetics. Pharm. Chem. J..

[13] Bennett JL, Mackie AL, Park Y, Gagnon GA (2018). Advanced oxidation processes for treatment of 17β-Estradiol and its metabolites in aquaculture wastewater. Aquacult. Eng..

[14] Tan WK, Cheah SC, Parthasarathy S, Rajesh R, Pang CH, Manickam S (2021). Fish pond water treatment using ultrasonic cavitation and advanced oxidation processes. Chemosphere.

[15] Chen S, Yu J, Wang H, Yu H, Quan X (2015). A pilot-scale coupling catalytic ozonation–membrane filtration system for recirculating aquaculture wastewater treatment. Desalination.

[16] Webb J, Quintã R, Papadimitriou S, Norman L, Rigby M, Thomas D, Le Vay L (2012). Halophyte filter beds for treatment of saline wastewater from aquaculture. Water Res..

[17] Calderini ML, Stevčić Č, Taipale S, Pulkkinen K (2021). Filtration of Nordic recirculating aquaculture system wastewater: Effects on microalgal growth, nutrient removal, and nutritional value. Algal Res..

[18] Ferreira CI, Calisto V, Otero M, Nadais H, Esteves VI (2017). Removal of tricaine methanesulfonate from aquaculture wastewater by adsorption onto pyrolysed paper mill sludge. Chemosphere.

[19] Yu R, Yu X, Xue B, Liao J, Zhu W, Tian S (2020). Adsorption of chlortetracycline from aquaculture wastewater using modified zeolites. J. Environ. Sci. Health Part A.

[20] Yu R, Yu X, Xue B, Liao J, Zhu W, Fu J (2021). Adsorption of oxytetracycline from aquaculture wastewater by modified carbon nanotubes: Kinetics, isotherms and thermodynamics. Fuller. Nanotub. Carbon Nanostruct..

[21] Xu J, Du Y, Qiu T, Zhou L, Li Y, Chen F, Sun J (2021). Application of hybrid electrocoagulation–filtration methods in the pretreatment of marine aquaculture wastewater. Water Sci. Technol..

[22] Igwegbe CA, Onukwuli OD, Ighalo JO, Umembamalu CJ (2021). Electrocoagulation–flocculation of aquaculture effluent using hybrid iron and aluminium electrodes: A comparative study. Chem. Eng. J. Adv..

[23] Igwegbe CA, Onukwuli OD, Onyechi PC (2019). Optimal route for turbidity removal from aquaculture wastewater by electrocoagulation–flocculation process. J. Eng. Appl. Sci..

[24] Li B, Jia R, Hou Y, Zhang C, Zhu J, Ge X (2021). The sustainable treatment effect of constructed wetland for the aquaculture effluents from blunt snout bream (*Megalobrama amblycephala*) farm. Water.

[25] Sindilariu P-D, Schulz C, Reiter R (2007). Treatment of flow-through trout aquaculture effluents in a constructed wetland. Aquaculture.

[26] Ahmad AL, Chin JY, Harun MHZM, Low SC (2022). Environmental impacts and imperative technologies towards sustainable treatment of aquaculture wastewater: A review. J. Water Process Eng..

[27] Heinen J, Hankins J, Adler P (1996). Water quality and waste production in a recirculating trout-culture system with feeding of a higher-energy or a lower-energy diet. Aquac. Res..

[28] Enduta A, Jusoh A, Ali NA, Wan Nik W (2011). Nutrient removal from aquaculture wastewater by vegetable production in aquaponics recirculation system. Desalin. Water Treat..

[29] Endut A, Lananan F, Abdul Hamid SH, Jusoh A, Wan Nik WN (2016). Balancing of nutrient uptake by water spinach (*Ipomoea aquatica*) and mustard green (*Brassica juncea*) with nutrient production by African catfish (*Clarias gariepinus*) in scaling aquaponic recirculation system. Desalin. Water Treat..

[30] Ebeling JM, Ogden SR, Sibrell PL, Rishel KL (2004). Application of chemical coagulation aids for the removal of suspended solids (TSS) and phosphorus from the microscreen effluent discharge of an intensive recirculating aquaculture system. N. Am. J. Aquac..

[31] Alnawajha MM, Kurniawan SB, Imron MF, Abdullah SRS, Hasan HA, Othman AR (2022). Plant-based coagulants/flocculants: characteristics, mechanisms, and possible utilization in treating aquaculture effluent and benefiting from the recovered nutrients. Environ. Sci. Pollution Res..

[32] Karam A, Bakhoum ES, Zaher K (2021). Coagulation/flocculation process for textile mill effluent treatment: experimental and numerical perspectives. Int. J. Sustain. Eng..

[33] Abujazar MSS, Karaağaç SU, Amr SSA, Alazaiza MY, Bashir MJ (2022). Recent advancement in the application of hybrid coagulants in coagulation–flocculation of wastewater: A review. J. Clean. Prod..

[34] Sahu O, Chaudhari P (2013). Review on chemical treatment of industrial waste water. J. Appl. Sci. Environ. Manag..

[35] Binnie C, Kimber M, Smethurst G (2002). Basic Water Treatment.

[36] Miller SM, Fugate EJ, Craver VO, Smith JA, Zimmerman JB (2008). Toward understanding the efficacy and mechanism of *Opuntia* spp. as a natural coagulant for potential application in water treatment. Environ. Sci. Technol..

[37] Shan TC, Matar MA, Makky EA, Ali EN (2017). The use of Moringa oleifera seed as a natural coagulant for wastewater treatment and heavy metals removal. Appl. Water Sci.

[38] Menkiti M, Ejimofor M, Ezemagu I, Uddameri V (2016). Turbid-metric approach on the study of adsorptive component of paint effluent coagulation using snail shell extract. Arab. J. Sci. Eng..

[39] Teh CY, Wu TY, Juan JC (2014). Potential use of rice starch in coagulation–flocculation process of agro-industrial wastewater: treatment performance and flocs characterization. Ecol. Eng..

[40] Bratby J (2016). Coagulation and Flocculation in Water and Wastewater Treatment.

[41] Nharingo T, Zivurawa M, Guyo U (2015). Exploring the use of cactus Opuntia ficus indica in the biocoagulation–flocculation of Pb (II) ions from wastewaters. Int. J. Environ. Sci. Technol..

[42] Okoro BU, Sharifi S, Jesson MA, Bridgeman J (2021). Natural organic matter (NOM) and turbidity removal by plant-based coagulants: A review. J. Environ. Chem. Eng..

[43] Ovuoraye PE, Ugonabo VI, Nwokocha GF (2021). Optimization studies on turbidity removal from cosmetics wastewater using aluminum sulfate and blends of fishbone. SN Appl. Sci..

[44] Onyechi KK, Igwegbe CA (2019). Determination of shelf life of Picralima nitida, ciprofloxacin and pefloxacin using bio-based concentration-activity relationship technique. Asian J. Res. Med. Pharm. Sci.

[45] Onyechi KK, Igwegbe CA (2019). Shelf life assessment of *Picralima nitida* and *Glibenclamide* using bio-based dose–response relationship method. Asian J. Res. Med. Pharmaceut. Sci..

[46] Igwegbe CA, Onukwuli OD, Ighalo JO, Menkiti MC (2021). Bio-coagulation–flocculation (BCF) of municipal solid waste leachate using picralima nitida extract: RSM and ANN modelling. Curr. Res. Green and Sustain. Chem..

[47] Igwegbe, C. A. *Evaluation of bio- and electro- coagulants' activities on Fish pond wastewater and Solid waste leachate. *In *Department of Chemical Engineering* (Nnamdi Azikiwe University, Awka, Nigeria, 2019).

[48] APHA. *Standard Methods for the Examination of Water and Wastewater.* In *American Public Health Association (APHA), American Water Works Association (AWWA) and Water Pollution Control Federation (WPCF),* Washington DC. 18th Ed. (1992).

[49] APHA. *Standard Methods for the Examination of Water and Wastewater*. 19th edn (1995).

[50] APHA. *Standard Methods for the Examination of Water and Wastewater*. 20th edn (APHA, AWWA, and WEF, 1998).

[51] EPA. *Method 180.1: Determination of turbidity by nephelometry.* (Environmental Monitoring Systems Laboratory Office of Research and Development U.S. Environmental Protection Agency, 1993).

[52] Smoluchowski, M. *Versuch einer mathematischen Theorie der Koagulationskinetik kolloider Lösungen* (1917).

[53] Emembolu LN, Igwegbe CA, Ugonabo VI (2016). Effect of natural biomass treatment on vegetable oil industry effluent via coag-flocculation. Saudi J. Eng. Technol..

[54] Ugonabo, V. I., Emembolu, L. N., Igwegbe, C. A. & Olaitan, S. A. Optimal evaluation of coag-flocculation factors for refined petroleum wastewater using plant extract. In *International Conference 2016 Proceedings* (FACULTY OF ENGINEERING, UNIZIK, 2016).

[55] Menkiti M, Igbokwe P, Ugodulunwa F, Onukwuli O (2008). Rapid coagulation/flocculation kinetics of coal effluent with high organic content using blended and unblended chitin derived coagulant (CSC). Res. J. Appl. Sci..

[56] Igwegbe CA, Onukwuli OD (2019). Removal of total dissolved solids (TDS) from aquaculture wastewater by coagulation–flocculation process using *Sesamum indicum* extract: Effect of operating parameters and coagulation–flocculation kinetics. Pharm. Chem. J..

[57] Menkiti MC, Nwoye CI, Onyechi CA, Onukwuli OD (2011). Factorial optimization and kinetics of coal washery effluent coag-flocculation by Moringa oleifera seed biomass. Adv. Chem. Eng. Sci..

[58] Fridrikhsberg, D. A. *A Course in Colloid Chemistry*. (Imported Pubn, 1986).

[59] Ugonabo VI, Emembolu LN, Igwegbe CA (2016). Bio-coag-flocculation of refined petroleum wastewater using plant extract: A turbidimeric approach. Int. J. Eng. Res. Technol..

[60] Igwegbe, C. A., Ighalo, J. O., Ghosh, S., Ahmadi, S. & Ugonabo, V. I. *Pistachio (Pistacia vera) waste as adsorbent for wastewater treatment: A review.* Biomass Convers. Biorefin. 1–19 (2021).

[61] Menkiti MC, Ejimofor MI (2016). Experimental and artificial neural network application on the optimization of paint effluent (PE) coagulation using novel Achatinoidea shell extract (ASE). J. Water Process Eng..

[62] Okey-Onyesolu C, Onukwuli O, Ejimofor M, Okoye C (2020). Kinetics and mechanistic analysis of particles decontamination from abattoir wastewater (ABW) using novel Fish Bone Chito-protein (FBC). Heliyon.

[63] Mageshkumar M, Karthikeyan R (2016). Modelling the kinetics of coagulation process for tannery industry effluent treatment using Moringa oleifera seeds protein. Desalin. Water Treat..

[64] Cai X, Tyagi S (2014). Development of a product life-cycle cost estimation model to support engineering decision-making in a multi-generational product development environment. J. Cost Anal. Parametr..

[65] Beltrán-Heredia J, Sánchez-Martín J, Gómez-Muñoz C (2012). Performance and characterization of a new tannin-based coagulant. Appl. Water Sci..

[66] Packham R (1972). The laboratory evaluation of polyelectrolyte flocculants. Br. Polym. J..

[67] Lagergren S, Svenska BK (1898). On the theory of so-called adsorption of dissolved substances. R. Swed. Acad. Sci. Doc..

[68] Oba SN, Ighalo JO, Aniagor CO, Igwegbe CA (2021). Removal of ibuprofen from aqueous media by adsorption: A comprehensive review. Sci. Total Environ..

[69] Ho YS, McKay G (1999). Pseudo-second order model for sorption processes. Process Biochem..

[70] Aniagor CO, Igwegbe CA, Ighalo JO, Oba SN (2021). Adsorption of doxycycline from aqueous media: A review. J. Mol. Liq..

[71] Zhang Y, Cheng L, Ji Y (2022). A novel amorphous porous biochar for adsorption of antibiotics: Adsorption mechanism analysis via experiment coupled with theoretical calculations. Chem. Eng. Res. Des..

[72] Li C, Gao Y, Li A, Zhang L, Ji G, Zhu K, Wang X, Zhang Y (2019). Synergistic effects of anionic surfactants on adsorption of norfloxacin by magnetic biochar derived from furfural residue. Environ. Pollut..

[73] Ahmadi S, Igwegbe CA, Rahdar S, Asadi Z (2019). The survey of application of the linear and nonlinear kinetic models for the adsorption of nickel (II) by modified multi-walled carbon nanotubes. Appl. Water Sci..

[74] Banerjee S, Dubey S, Gautam RK, Chattopadhyaya M, Sharma YC (2017). Adsorption characteristics of alumina nanoparticles for the removal of hazardous dye, Orange G from aqueous solutions. Arab. J. Chem..

[75] Hubbe MA, Rojas OJ (2008). Colloidal stability and aggregation of lignocellulosic materials in aqueous suspension: A review. BioResources.

[76] Zhang Z, Xia S, Zhao J, Zhang J (2010). Characterization and flocculation mechanism of high efficiency microbial flocculant TJ-F1 from Proteus mirabilis. Colloids Surf. B.

[77] Awang NA, Aziz HA (2012). Hibiscus rosa-sinensis leaf extract as coagulant aid in leachate treatment. Appl. Water Sci..

[78] Misau IM, Yusuf AA (2016). Characterization of water melon seed used as water treatment coagulant. J. Adv. Stud. Agric. Biol. Environ. Sci..

[79] Bhandari VM, Ranade VV (2014). Advanced physico-chemical methods of treatment for industrial wastewaters. Industrial Wastewater Treatment, Recycling and Reuse.

[80] Loganathan P, Gradzielski M, Bustamante H, Vigneswaran S (2020). Progress, challenges, and opportunities in enhancing NOM flocculation using chemically modified chitosan: a review towards future development. Environ. Sci. Water Res. Technol..

[81] Fedala N, Lounici H, Drouiche N, Mameri N, Drouiche M (2015). RETRACTED: Physical Parameters Affecting Coagulation of Turbid Water with Opuntia ficus-indica Cactus.

[82] Cruz D, Pimentel M, Russo A, Cabral W (2020). Charge neutralization mechanism efficiency in water with high color turbidity ratio using aluminium sulfate and flocculation index. Water.

[83] Obiora-Okafo I, Onukwuli O, Eli-Chukwu N (2020). Evaluation of bio-coagulants for colour removal from dye synthetic wastewater: Characterization, adsorption kinetics, and modelling approach. Water SA.

[84] Chowdhury M, Mostafa M, Biswas TK, Saha AK (2013). Treatment of leather industrial effluents by filtration and coagulation processes. Water Resour. Ind..

[85] Shak KPY, Wu TY (2014). Coagulation–flocculation treatment of high-strength agro-industrial wastewater using natural Cassia obtusifolia seed gum: treatment efficiencies and flocs characterization. Chem. Eng. J..

[86] Zuki, N.M., N. Ismail, and F.M. Omar. *Evaluation of zeta potential and particle size measurements of multiple coagulants in semiconductor wastewater*. In *AIP Conference Proceedings* (AIP Publishing LLC, 2019).

[87] Beltrán-Heredia J, Sánchez-Martín J, Delgado-Regalado A, Jurado-Bustos C (2009). Removal of Alizarin Violet 3R (anthraquinonic dye) from aqueous solutions by natural coagulants. J. Hazard. Mater..

[88] Ejimofor M, Ezemagu I, Ugonabo V, Nnaji P, Anadebe V, Diyoke C, Menkiti M (2022). Adsorption kinetics, mechanistic, isotherm and thermodynamics study of petroleum produced water coagulation using novel Egeria radiate shell extract (ERSE). J. Indian Chem. Soc..

[89] Okolo B, Nnaji P, Menkiti M, Onukwuli O (2015). A kinetic investigation of the pulverized okra pod induced coag-flocculation in treatment of paint wastewater. Am. J. Anal. Chem..

[90] Phalakornkule C, Mangmeemak J, Intrachod K, Nuntakumjorn B (2010). Pretreatment of palm oil mill effluent by electrocoagulation and coagulation. Science Asia.

[91] Marriott NG, Robertson G (1997). Essentials of Food Sanitation.

[92] Bhatia S, Othman Z, Ahmad AL (2007). Pretreatment of palm oil mill effluent (POME) using *Moringa oleifera* seeds as natural coagulant. J. Hazard. Mater..

[93] Ugonabo IV, Onukwuli O, Ezechukwu C (2020). Deturbidization of pharmaceutical industry wastewater using natural coagulant: Response surface methodology applied. Int. J. Progress. Sci. Technol..

[94] Ovuoraye PE, Ugonabo VI, Tahir A, Balogun PA (2022). Kinetics-driven Coagulation treatment of petroleum refinery effluent using Land snail shells: An empirical approach to Environmental sustainability. Clean. Chem. Eng..

[95] WST. *About Coagulation and Flocculation.* 1–10 (Information Bulletins, Water Specialist Technology (WST), 2005).

[96] Ejikeme EM, Ejikeme PCN, Offia K (2020). Coagulation Kinetics for bakery wastewater treatment using Vigna Subterranea husk as coagulant. Researcher.

[97] Hunter R (1993). Introduction to Modern Colloid Science.

[98] Menkiti MC, Ezemagu IG (2015). Sludge characterization and treatment of produced water (PW) using Tympanotonus fuscatus coagulant (TFC). Petroleum.

[99] Ifeanyi U, Chukwudi MM, Okechukwu OD (2012). Effect of coag-flocculation kinetics on telfairia occidentalis seed coagulant (TOC) in pharmaceutical wastewater. Int J Multidisciplin Sci Eng.

[100] de Oliveira Reis, G. *Study of the Mechanism of Acid Coagulation of Hevea Latex and of the Rheological Properties of Resulting Gels* (Université de Montpellier, 2015).

[101] Menkiti, M., Nnaji, P., & Onukwuli, O. *Coag-Flocculation Kinetics and Functional Parameters Response of Periwinkle Shell Coagulant (PSC) to pH Variation in Organic Rich Coal Effluent Medium.*

[102] Menkiti M, Onyechi C, Onukwuli O (2011). Evaluation of perikinetics compliance for the coag-flocculation of brewery effluent by Brachystegia eurycoma seed extract. Int. J. Multidiscip. Sci. Eng..

[103] Obiora-Okafo IA, Onukwuli OD, Igwegbe CA, Onu CE, Omotioma M (2022). Enhanced performance of natural polymer coagulants for dye removal from wastewater: Coagulation kinetics, and mathematical modelling approach. Environ. Processes.

[104] Park S, Kruis F, Lee K, Fissan H (2002). Evolution of particle size distributions due to turbulent and Brownian coagulation. Aerosol Sci. Technol..

[105] Abdulahi, M. B., Adeoye, P. A. & Amao, O. S. Evaluation of coagulation efficiency of Moringa oleifera extract and alum on fish pond wastewater.In *37th Annual Conference and Annual General Meeting of NIAE, MInna* (2016).

[106] Ohale PE, Onu CE, Ohale NJ, Oba SN (2020). Adsorptive kinetics, isotherm and thermodynamic analysis of fishpond effluent coagulation using chitin derived coagulant from waste Brachyura shell. Chem. Eng. J. Adv..

[107] Chung Y-C, Li Y-H, Chen C-C (2005). Pollutant removal from aquaculture wastewater using the biopolymer chitosan at different molecular weights. J. Environ. Sci. Health Part A.

[108] Kurniawan SB, Imron MF, Abdullah SRS, Othman AR, Purwanti IF, Hasan HA (2022). Treatment of real aquaculture effluent using bacteria-based bioflocculant produced by *Serratia marcescens*. J. Water Process Eng..

[109] Ahmad A, Abdullah SRS, Hasan HA, Othman AR, Ismail NI (2021). Plant-based versus metal-based coagulants in aquaculture wastewater treatment: Effect of mass ratio and settling time. J. Water Process Eng..

[110] Dong C, Zhang C, Wang B (2003). Integration of green quality function deployment and fuzzy multi-attribute utility theory-based cost estimation for environmentally conscious product development. Int. J. Environ. Conscious Des. Manuf..

